# The Emergence of Quinone Methides in Asymmetric Organocatalysis

**DOI:** 10.3390/molecules200711733

**Published:** 2015-06-25

**Authors:** Lorenzo Caruana, Mariafrancesca Fochi, Luca Bernardi

**Affiliations:** Department of Industrial Chemistry “Toso Montanari” and INSTM RU of Bologna, Alma Mater Studiorum, University of Bologna, V. Risorgimento 4, 40136 Bologna, Italy; E-Mail: lorilu@hotmail.it

**Keywords:** asymmetric catalysis, conjugate addition, cycloaddition, organocatalysis, phenol, quinone methide

## Abstract

Quinone methides (QMs) are highly reactive compounds that have been defined as “elusive” intermediates, or even as a “synthetic enigma” in organic chemistry. Indeed, there were just a handful of examples of their utilization in catalytic asymmetric settings until some years ago. This review collects organocatalytic asymmetric reactions that employ QMs as substrates and intermediates, from the early examples, mostly based on stabilized QMs bearing specific substitution patterns, to more recent contributions, which have dramatically expanded the scope of QM chemistry. In fact, it was only very recently that the generation of QMs *in situ* through strategies compatible with organocatalytic methodologies has been realized. This tactic has finally opened the gate to the full exploitation of these unstable intermediates, leading to a series of remarkable disclosures. Several types of synthetically powerful asymmetric addition and cycloaddition reactions, applicable to a broad range of QMs, are now available.

## 1. Introduction

**Scheme 1:**
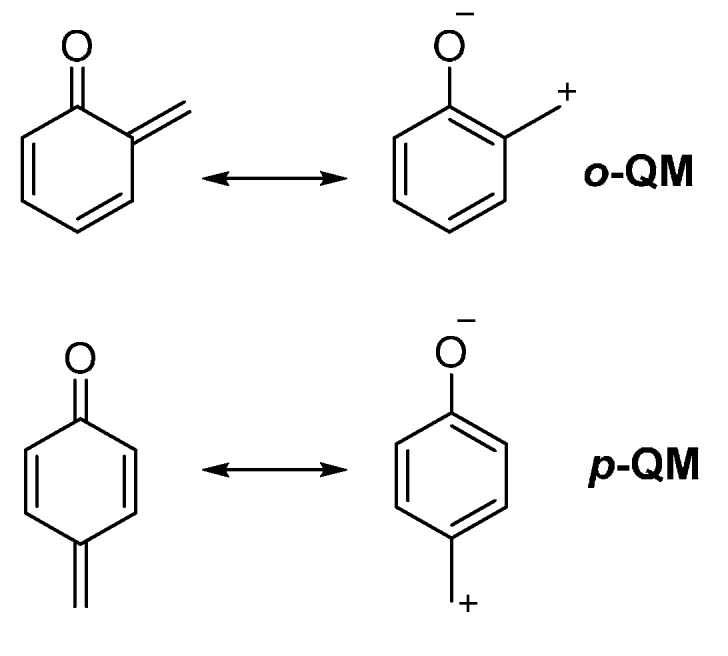
*ortho*-Quinone methide (*o*-QM) and *para*-quinone methide (*p*-QM): Resonance structures.

Quinone methides (QMs) display a cyclohexadiene core featuring an exocyclic alkylidene and a carbonyl residue, mainly disposed at *ortho* or *para* position. The 1,2- and 1,4-quinone methides (namely *ortho*- and *para*-quinone methides, *o*-QM and *p*-QM, respectively) are formally neutral molecules; however, the zwitterionic aromatic resonance structures are highly relevant, rendering these molecules highly polarized and thus reactive at those sites (Scheme 1).

**Scheme 2:**
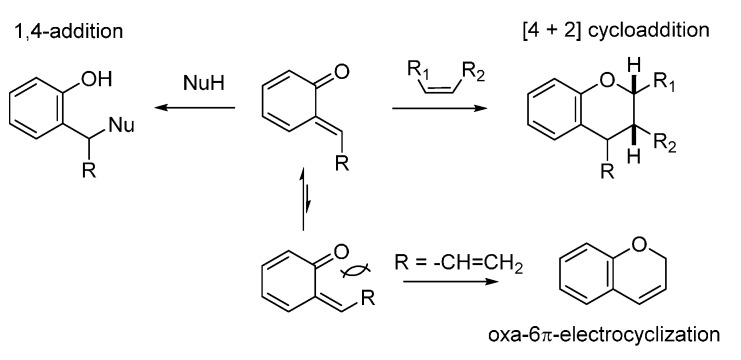
The three typical reaction pathways of *o*-QMs.

QMs can be considered as Michael acceptors even if, compared to standard enones, their reactivity is much more pronounced. In fact, while conjugate addition of nucleophiles to simple enones results in a small decrease in the stability of the system due to the loss of conjugation and simultaneous formation of enol intermediates, addition of nucleophiles to QMs results in a large increase in the π-stabilization energy due to the formation of a fully aromatic ring; this provides a considerable driving force that exceedingly enhances the reactivity of QMs [1]. While *p*-QMs react only in 1,6-conjugate additions, the arrangement of the carbonyl oxygen and the exocyclic alkylidene in *o*-QMs gives them a richer chemistry. The reactivity of *o*-QMs is expressed by three typical reaction pathways, involving not only the 1,4-addition of various nucleophiles, but also [4 + 2] cycloadditions with 2π partners and oxa-6π-electrocyclizations (Scheme 2). The two latter pathways provide direct access to benzopyran structures, common scaffolds in natural products. Of great relevance in stereoselective synthesis, the configuration of the exocyclic olefin in *o*-QMs is controlled by steric effects, the *E*-isomer being favored in 5-unsubstituted *o*-QM due to the steric repulsion between the oxygen and the R substituent. However, due to the contribution of the zwitterionic resonance structure, this double bond shows a more pronounced fluxional behavior compared to a simple olefin, allowing the enone system to reach the required configuration for undergoing the 6π-electrocyclization process.

From a historical perspective, *o*-QMs were first invoked by Fries and Kann in 1907 [2], but only decades later more direct evidence of their existence was collected, first by using low-temperature IR spectroscopy [3] and more recently by X-ray diffraction experiments in which *o*-QMs were entrapped in an iridium complex [4]. Despite their high reactivity, QMs are relatively common moieties in biological settings. QMs are often proposed as biosynthetic intermediates, and several natural compounds contain these moieties. More importantly, the biological activity/toxicity featured by many natural/synthetic molecules is attributed to the reactivity of (transiently generated) QMs subunits, acting as powerful alkylating agents towards biological targets. These observations have spurred very active research areas dealing with the control/sensing of biological processes through the generation of reactive QMs *in situ* [5,6].

In contrast, the marked reactivity of QMs has been generally seen as a deterrent to their employment in organic synthesis. In their key review published in 2002, Van De Water and Pettus have called *o*-QMs as “intermediates underdeveloped and underutilized in organic synthesis”, or even as a “synthetic enigma” [7]. A more recent review by Singh defines *o*-QM as a “highly reactive, ephemeral and versatile intermediate” [8]. Although their relevance as biosynthetic intermediates has prompted the disclosure of a number of natural product syntheses based on QMs [9], most QMs tend to react through undesired pathway (e.g., dimerization) or undergo decomposition, and are non-isolable, thus justifying the above definitions. In fact, the full engagement of these species in organic synthesis is necessarily linked with the disclosure of general platforms for their generation *in situ* from simple precursors and under manageable conditions. In a progression that has been recently called by Pettus as a “domestication” of these species [10], several approaches for the *in situ* generation of *o*-QMs have been disclosed, wherein the method of generation dictates the manner in which the intermediate can be utilized.

Perhaps due to the reluctance of organic chemists in exploring such “enigmatic” intermediates, examples of QMs in catalytic asymmetric settings have been scarce until very recently. Initial studies mostly dealt with stabilized *o*-QMs, wherein multiple electron-donating substituents at the electron-poor triene system make their isolation possible, carrying along obvious limitations in substrate scope. Important disclosures by Sigman based on palladium activation of 2-hydroxystyrenes giving *o*-QM intermediates, and amenable to enantioselective versions, are also worth mentioning [11]. However, it was only starting from 2012 that several key contributions showed how the ingenious combination of organocatalytic strategies with the *in situ* generation of *o*-QMs from various precursors could open the gate to the full exploitation of these intermediates in asymmetric catalytic transformations. Furthermore, *p*-QMs started to be engaged in catalytic asymmetric processes. Based on our interest in the area, we thought it timely to collect and summarize in this focused review all organocatalytic asymmetric reactions based on QMs, starting from early examples until more recent developments.

## 2. Catalytic Asymmetric Reactions with Stabilized *o*-QMs

**Scheme 3:**
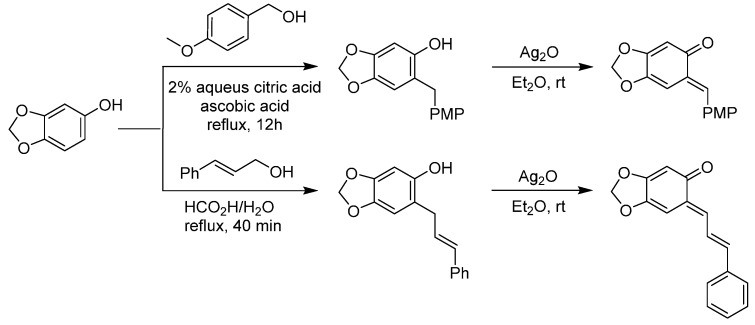
Synthesis of stabilized *o*-QMs. PMP = *p*-methoxyphenyl.

*o*-QMs have attracted considerable attention because of their intriguing structure and properties. *o*-QMs serve as important intermediates in a variety of biological pathway and enable the straightforward construction of products of broad synthetic utility. Typically *o*-QMs are generated *in situ* under Lewis acidic, basic, thermal or photochemical conditions. Less usual is the synthesis of stabilized and isolable *o*-QMs whose reactivity is controllable. As early as 1977, Jurd firstly synthetized electron-rich *o*-QMs in two steps from sesamol by condensation with the appropriate alcohol (4-methoxybenzyl alcohol and cinnamyl alcohol, respectively), followed by Ag_2_O oxidation (Scheme 3) [12]. The possibility of working with isolable substrates has allowed initial breakthroughs in the utilization of *o*-QMs for organocatalytic asymmetric reactions, as summarized in the next paragraphs.

**Scheme 4:**
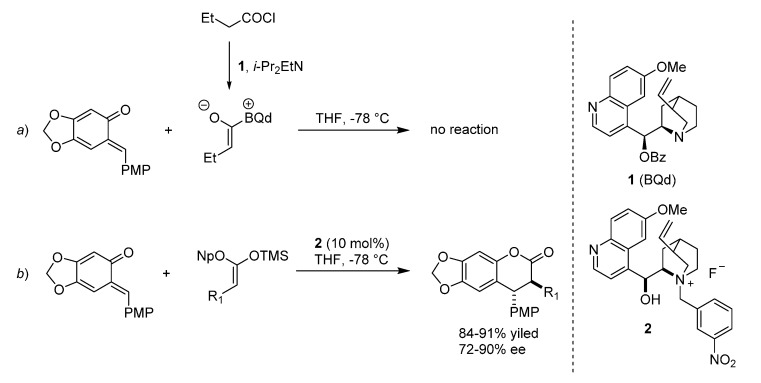
Synthesis of 3,4-dihydrocoumarin derivatives utilizing silyl ketene acetal. Np = 2-naphthyl.

The PMP derived *o*-QM was chosen as a test substrate for the formal catalytic [4 + 2] cycloaddition with ketene enolate by Lectka for the construction of 3,4-dihydrocoumarin derivatives with contiguous stereocenters (Scheme 4) [13]. Firstly, the *o*-QM was treated with butyryl chloride and the kinetic base/chiral catalyst *O*-benzoylquinidine (**1**, BQd), in the presence of the thermodynamic Hunig’s base to neutralize the hydrogen chloride formed in the reaction. However, these conditions produced no product and the authors suggested that the high electron density on *o*-QM was not compatible with the mild nucleophilicity of the zwitterionic ketene enolate (Scheme 4a).

**Scheme 5:**
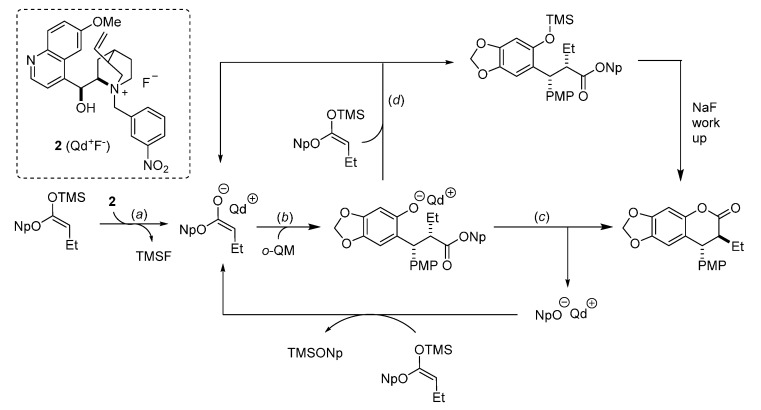
Possible reaction pathways for enantioselective chiral ammonium fluoride **2** initiated [4 + 2] cycloaddition reaction.

Therefore, silyl ketene acetal was used as the ester enolate precursor (Scheme 4b). The reaction of *o*-QM with a silyl ketene acetal in THF, in the presence of a chiral ammonium fluoride precatalyst **2** afforded the desired cycloaddition product with 80% ee (R_1_ = Et). The versatility of the methodology was proved by testing a selection of ketene acetals bearing both aliphatic and aryl substituents. The formal [4 + 2] cycloadducts were formed in high yields and with good enantioselectivities. The authors showed that the precatalyst effect on the enantioselectivity of the reaction was not dictated by steric bulk, but rather by electronic effects. The reaction proceeds as depicted in Scheme 5, by fluoride ion desilylation of the ketene acetal forming chiral ion-paired ketene enolate (*a*). The ketene enolate then regioselectively alkylates the *o*-QM with the restoration of aromaticity as the driving force (*b*). Lactonization (*c*) forms the desired cycloadduct with release of the quinidinium 2-naphtoxide, which can in its turn desilylate a new molecule of silyl ketene acetal closing the catalytic cycle. Alternatively, it is the phenoxide that can react with the silyl ketene acetal substrate, giving the same quinidinium enolate and the corresponding *O*-silylated derivative (*d*). A sodium fluoride wash in the workup ensures complete conversion to the desired lactone.

**Scheme 6:**
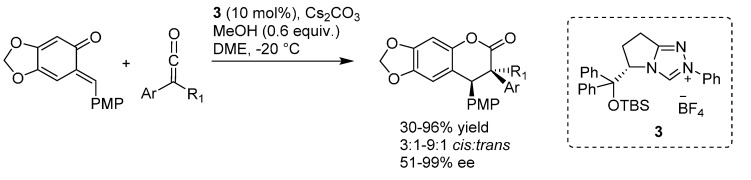
Catalytic enantioselective synthesis of 3,4-dihydrocoumarins via [4 + 2] cycloaddition between ketenes and a stabilized *o*-QM catalyzed by an NHC.

Shortly after, Ye reported that *N*-heterocyclic carbenes (NHC) derived from pyroglutamic acid efficiently catalyze the formal [4 + 2] cycloaddition of alkyl(aryl)ketenes with a stabilized *o*-QM [14]. The NHC was generated from the precursor **3** and Cs_2_CO_3_ as the base at room temperature, and used to afford the corresponding 3,4-dihydrocoumarin derivatives with two contiguous stereocenters in good yields and enantioselectivities (Scheme 6).

**Scheme 7:**
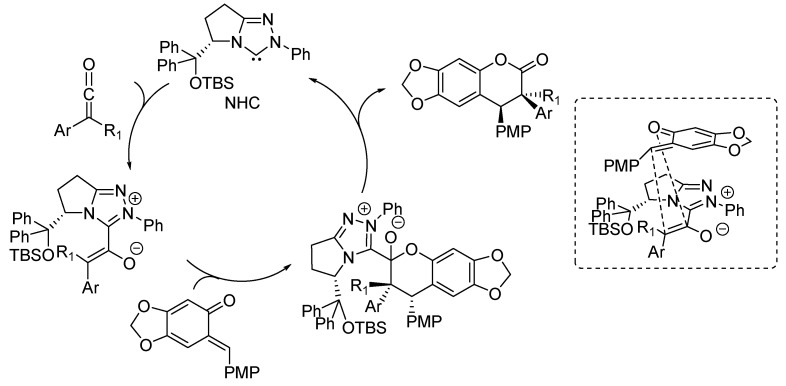
Proposed catalytic cycle and stereochemical model for the [4 + 2] cycloaddition between ketenes and an *o*-QM catalyzed by an NHC.

The authors proposed a catalytic cycle (Scheme 7) wherein the NHC generates an enolate by reaction with the ketene, which reacts with the *o*-QM via an inverse electron demand [4 + 2] cycloaddition. The resulting adduct furnishes the final product by fragmentation and regenerates the NHC catalyst.

**Scheme 8:**
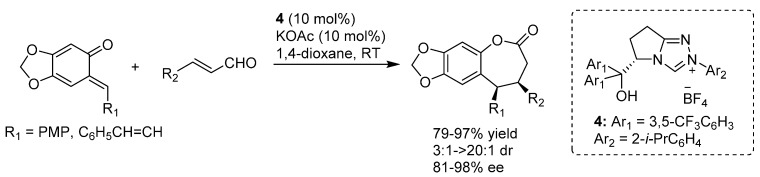
NHC-catalyzed [3 + 4] annulation of enals with stabilized *o*-QMs.

In 2013, the same laboratory described a different type of NHC-catalyzed cycloaddition reaction involving stabilized *o*-QMs, namely a [3 + 4] annulation with enals to give the corresponding benzo-ε-lactones (Scheme 8) [15]. Both β-aryl and β-alkyl enals furnished the corresponding benzo-ε-lactones in good to excellent yields and with moderate (for the β-aryl enals) to excellent (for the β-alkyl enals) diastereoselectivities. High to excellent enantioselectivities were achieved with the NHC catalyst, generated *in situ* from its precursor **4**, derived from l-pyroglutamic acid having a free hydroxyl group.

**Scheme 9:**
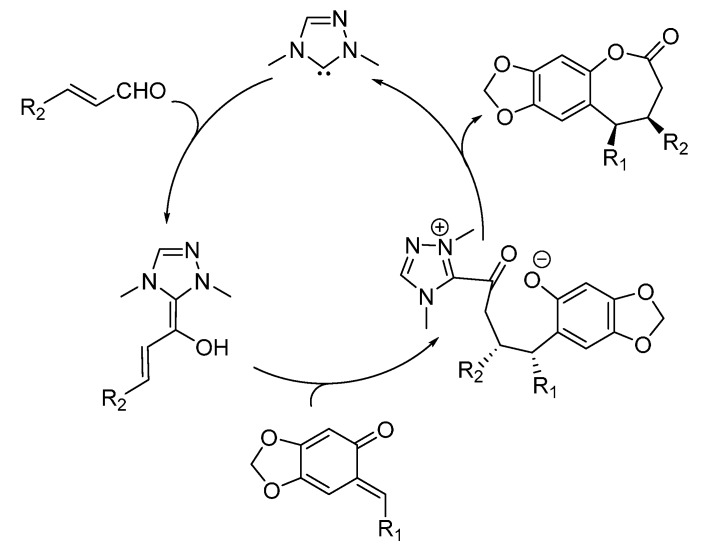
Proposed catalytic cycle for the [3 + 4] cycloaddition between *o*-QMs and enals.

To explain the obtained results, the authors proposed a catalytic cycle (Scheme 9) where the addition of the NHC to the enal forms the vinyl Breslow intermediate, which reacts with the *o*-QM by Michael addition. The obtained adduct gives an intramolecular lactonization to the final [3 + 4] annulation product and regenerates the NHC.

**Scheme 10:**
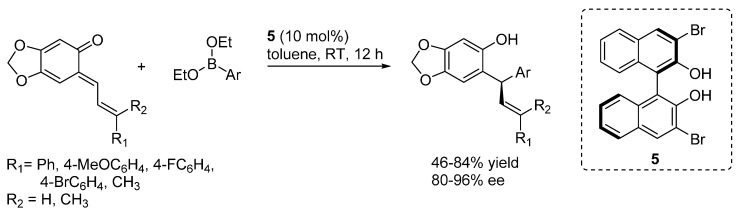
Enantioselective addition of aryl boronates to stabilized *o*-QMs.

A biphenol **5** catalyzed enantioselective asymmetric addition of aryl- or alkenylboronates to stabilized *o*-QMs was developed in 2012 by Luan and Schaus (Scheme 10) [16]. The boronate addition can be conducted under mild conditions leveraging the driving force for quinone methide rearomatization. Good yields and selectivities were observed with stabilized *o*-QMs bearing either electron-deficient or electron-rich vinyl group and with different aryl boronate nucleophiles. With the purpose of enlarging the scope of the reaction, the authors developed a procedure for the *in situ* generation of *o*-QMs that will be discussed in Section 3.2.

**Scheme 11:**
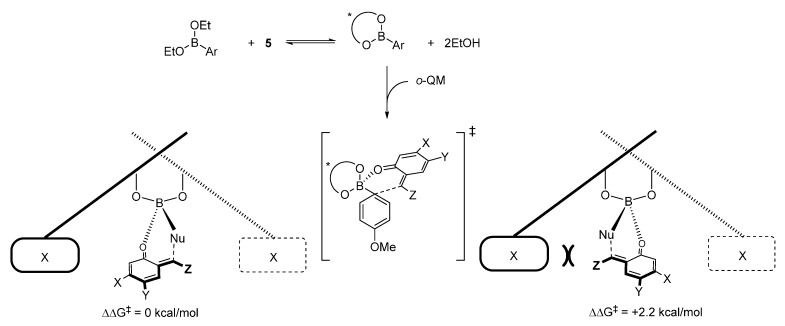
Competing TSs. Geometries B3LYP/6-31G**, single-point energies M06-2X/LACVP**.

Density functional theory calculations reported by Grayson and Goodman in 2015 suggested that asymmetric boronate addition to *o*-QMs proceeds via a Lewis acid catalyzed process through a closed six-membered *sofa like* transition structure (Scheme 11) [17]. The BINOL-derived catalyst undergoes an exchange process with the original ethoxide boronate ligands, delivering a vinyl boronate featuring a more pronounced Lewis acidity (*i.e.*, more reactive), which coordinates the *o*-QM and transfers the aryl ligand. This activation mode successfully accounts for the sense and level of enantioselectivity observed experimentally.

**Scheme 12:**
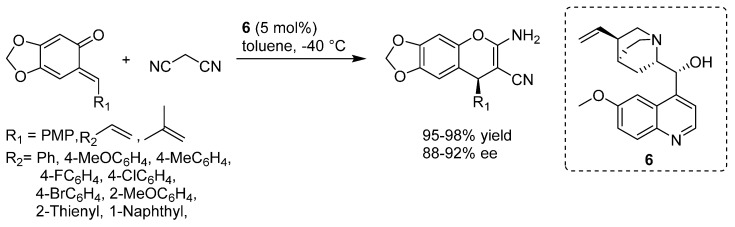
Quinine **6** catalyzed highly enantioselective [4 + 2] cycloaddition of *o*-QMs with malononitrile.

The quinine **6** catalyzed highly enantioselective formal [4 + 2] cycloaddition of *o*-QMs with malononitrile was presented by Han in 2015 for accessing optically active 2-amino-3-cyano-4*H*-chromenes, a class of potential anti-cancer agents, in excellent yields and enantioselectivities (Scheme 12) [18]. The reaction could be scaled up to 6 mmol without noticeable loss of yield and stereoselectivity.

## 3. Catalytic Asymmetric Reactions with *o*-QMs Generated *in Situ*

Although the generation of an *o*-QM *in situ* can in principle provide a more general approach, the disclosure of conditions compatible with both formation of a reactive *o*-QM and a catalytic asymmetric reaction appeared to be a very challenging task, unmet until very recently. Furthermore, ease of formation, reactivity and stability of these intermediates are highly dependent on the substitution pattern of their triene portion, with electron donating substituents facilitating their formation and isolation but at the same decreasing reactivity [19]. The most common method for the generation of *o*-QMs is the elimination of a stable molecule (e.g., water) from the benzylic position of 2-substituted phenols. In fact, although several other methods for the generation of *o*-QM are known (oxidations, olefinations, *etc.*), such type of elimination has certainly been the most common platform to form *o*-QMs *in situ* for asymmetric organocatalytic reactions, as summarized in the next sections. The conditions under which the *o*-QM is generated determine the type of catalysis that can be used in the asymmetric step, or, in a complementary perspective, the utilization of a type of catalysis mandates the conditions that can be used for the generation of the *o*-QM. The combinations of both acidic and basic conditions with appropriate catalysts have been implemented, thus allowing the productive engagement of a broad range of substrates in organocatalytic enantioselective reactions with *o*-QMs.

### 3.1. o-QMs Generated in Situ by Dehydration of Ortho-Hydroxybenzylic Alcohols under Brønsted Acid Conditions

**Scheme 13:**
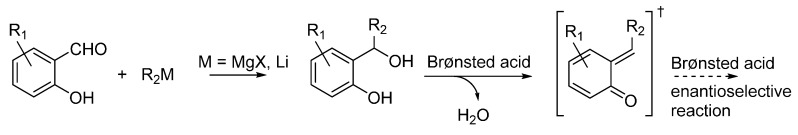
Preparation of *ortho*-hydroxybenzylic alcohols and reactions under acidic conditions.

The generation of *o*-QM by dehydration of *ortho*-hydroxybenzyl alcohols is perhaps the most common methodology exploited for the utilization of *o*-QM in chemical biology, wherein QMs bearing a terminal methylene group have been the most investigated. The position of the equilibrium existing between the alcohol and the *o*-QM, as well as the kinetics of *o*-QM formation, has been shown to be highly dependent on the conditions (especially pH) [20] and the substituents at the phenolic ring [19]. A general synthetic route to *ortho*-hydroxybenzylic alcohols is represented by the addition of organometallic reagents (Grignard and organolithium) to salicylaldehydes (Scheme 13). Useful protocols can be found in the papers highlighted in this section, which show how these benzylic alcohols can be engaged in Brønsted acid catalyzed transformations, wherein the acid promotes both the formation of the *o*-QM by dehydration and the ensuing enantioselective reaction.

**Scheme 14:**
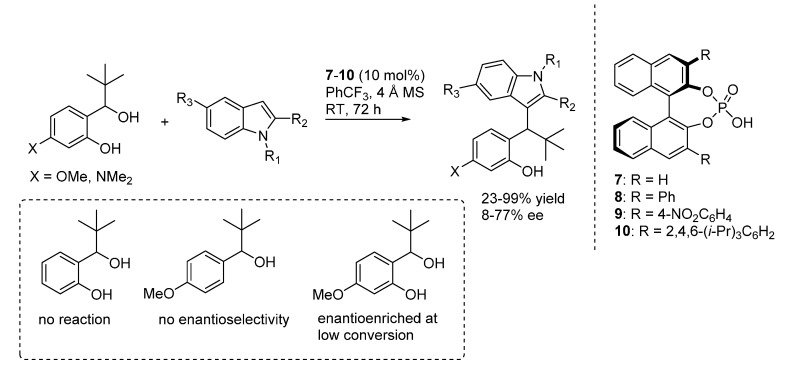
Addition of indoles to *o*-QMs generated from benzylic alcohols catalyzed by phosphoric acid catalysts **7**–**10**.

Speculating on the analogy between *o*-QMs and alkylideneindolenines, which can be formed by dehydration and activated upon the action of chiral phosphoric acid catalysts, Bach reported in 2011 that some *ortho*-hydroxybenzylic alcohols react with indoles in the presence of chiral catalysts **7**–**10**, to give the expected adducts through the intermediacy of *o*-QMs (Scheme 14) [21]. An electron donating substituent at the phenolic ring was found to be necessary for the reaction to proceed, presumably assisting formation of the intermediate. Whereas enantioselectivities were moderate at best, and no optimal catalyst giving uniformly good results with all substrates was found, this article represented the proof of concept of the viability of this approach, which other authors have later proven to be remarkably general. The intermediacy of an *o*-QM, formed upon the action of the acidic chiral phosphoric acid **7**–**10**, was demonstrated by the following control experiments: (i) a free phenol moiety was found to be mandatory for the reaction to proceed enantioselectively; (ii) the substitution at the alcohol was not stereospecific (*i.e.*, racemic or enantiopure benzylalcohol substrates gave comparable enantiomeric enrichments in the products); and (iii) in reaction which were not run to completion, the remaining benzylalcohol starting material was optically active.

**Scheme 15:**
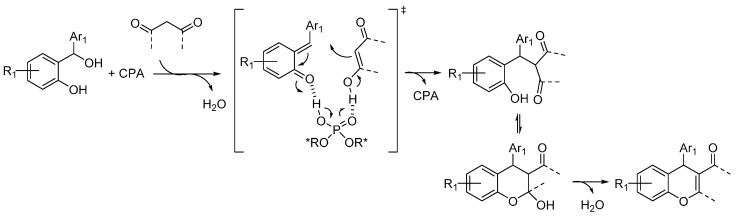
Addition of 1,3-dicarbonyl compounds to *o*-QMs catalyzed by CPA. CPA = Chiral Phosphoric Acid.

A catalytic enantioselective intramolecular cyclization involving dehydration of *ortho*-hydroxybenzylic alcohols in the presence of acidic catalysts was then reported by Rueping in 2011 [22]. However, the reaction was shown to proceed through a cationic intermediate, rather than through an *o*-QM implying a 6π-electrocyclization. While in 2013 a non-enantioselective example of a hetero-Diels-Alder cycloaddition involving an *o*-QM generated with this strategy was reported by Gharpure [23], it was not until 2014 that highly enantioselective examples of the combination of dehydration of 2-hydroxy benzylic alcohols and ensuing asymmetric additions promoted by chiral acidic catalysts appeared in the literature. Two nearly simultaneous reports by Schneider and by Rueping showed the viability of this approach in the addition of 1,3-carbonyl compounds, which upon intramolecular hemiacetalization and dehydration render 4*H*-chromenes or related polycyclic derivatives (Scheme 15) [24,25]. In both cases, the reactions appeared to be limited to 2-hydroxy benzhydryl alcohols as starting materials. Taking also into account computational studies by Freccero showing that hydrogen bonding to the QMs carbonyl can enhance their reactivity [26], and the commonly accepted mode of action of phosphoric acids, the catalysts were assumed to act in a bifunctional fashion. The acidic proton coordinates the carbonyl group of the *o*-QM and the Lewis basic P=O moiety the enolic proton of the nucleophile in the reaction transition state.

**Scheme 16:**
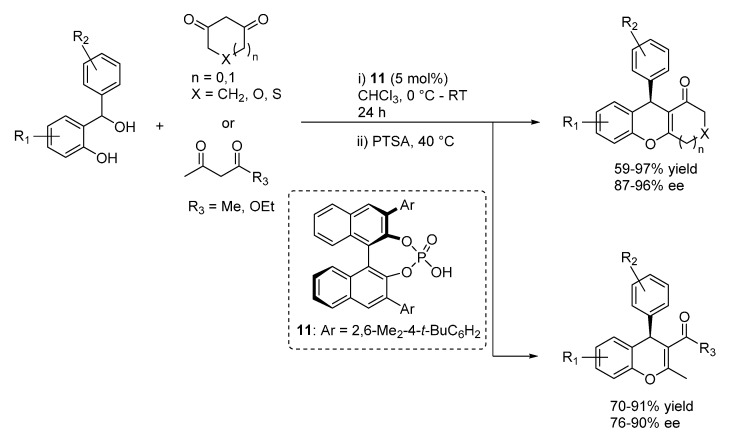
Addition of 1,3-dicarbonyl compounds to *o*-QMs catalyzed by phosphoric acid **11** developed by Schneider.

In more detail, Schneider reported the reaction with acetylacetone and cyclic 1,3-diketones featuring different ring sizes, and one example with a 3-ketoester, catalyzed by the BINOL derived phosphoric acid catalyst **11** (Scheme 16) [24]. A simple acidic treatment was performed to ensure full dehydration to the 4*H*-chromene products, which were obtained with very good results irrespective of the nucleophile and benzhydrylic alcohol employed.

**Scheme 17:**
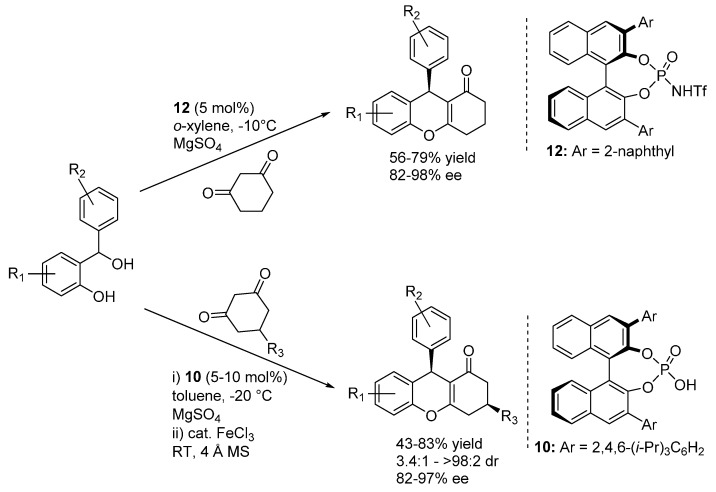
Addition of 1,3-dicarbonyl compounds to *o*-QMs catalyzed by phosphorimide **12** or phosphoric acid **10** developed by Rueping.

Rueping presented a similar catalytic reaction, using phosphorimide **12** or phosphoric acid **10** catalysts (Scheme 17) [25]; in the case of the more acidic phosphorimide **12**, acidic treatment after the reaction was not required, the catalytic product cyclized spontaneously to the 4*H*-chromene. While being limited to six membered diones, this report set great attention to a desymmetrization in the ensuing hemiacetalization and dehydration reactions, employing 5-substituted 1,3-cyclohexadiones as nucleophilic substrates and rendering tetrahydroxanthenes bearing two distant stereocenters with good diastereoselectivities.

**Scheme 18:**
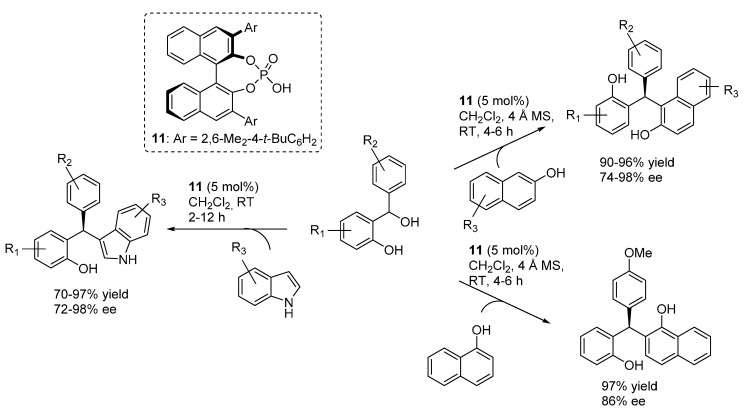
Addition of indoles and naphthols to *o*-QMs catalyzed by phosphoric acid catalyst **11**.

In a very short time, capitalizing on these disclosures, Schneider and co-workers extended this approach to other nucleophilic reaction partners. Reinvestigating reactions related to the low enantioselective examples reported by Bach (Scheme 14), they first prepared diarylindolylmethanes and triarylmethanes through the addition of indoles and naphthols (Scheme 18) [27]. These transformations exhibited a remarkable scope in terms of nucleophile partners, whereas once again appeared limited to benzhydrylic alcohols as *o*-QM precursors. Based on the low enantioselectivity obtained with an *N*-Boc indole, a usual bifunctional mode of action involving simultaneous coordination of *o*-QM and the indole or naphthol acidic proton, reminding the model depicted in Scheme 15 for 1,3-dicarbonyls, was put forward. Remarkably, the same catalyst **11** that proved to be optimal in the addition of 1,3-dicarbonyl compounds was found to be the best performing in the terms of enantioselectivity also for these unrelated Friedel-Crafts type reactions.

**Scheme 19:**
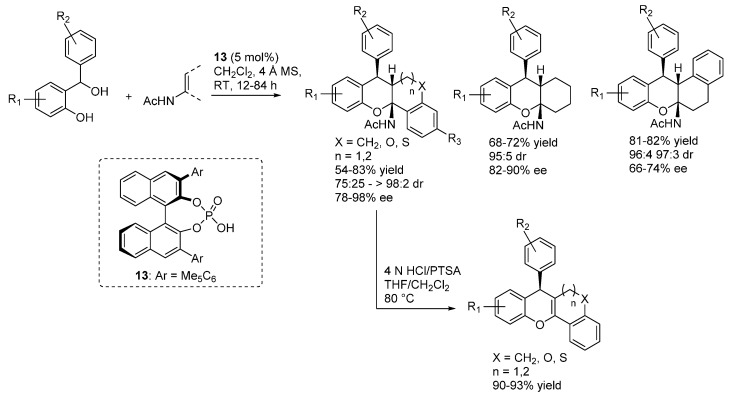
Addition of enamides to *o*-QMs catalyzed by phosphoric acid **13**.

A structurally distinct catalyst **13** was instead required for the catalytic asymmetric addition of enamides and enecarbamates, reported soon after by the same laboratory (Scheme 19) [28]. In this case, acetalization followed, resulting in a formal [4 + 2] cycloaddition. Acidic treatment on some of the adducts gave elimination of the amide, giving 4*H*-chromenes. These enamides thus acted as masked ketones, and allowed to prepare 4*H*-chromenes previously not accessible. In fact, whereas 1,3-dicarbonyls participate in the reaction thanks to their high enol content, direct utilization of mono-ketones was not possible.

**Scheme 20:**
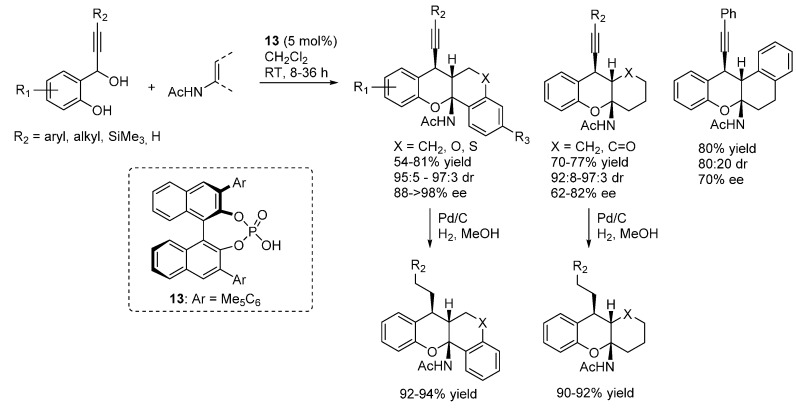
Addition of enamides to β-alkynyl substituted *o*-QMs catalyzed by phosphoric acid **13**.

In a subsequent publication, the same authors reported the important extension of this reaction to propargylic alcohols as starting materials, thus demonstrating the possibility of using *o*-QM intermediates different from β-aryl ones (Scheme 20) [29]. Furthermore, reduction of the triple bond ensured access 7-alkyl substituted xanthenes.

**Scheme 21:**
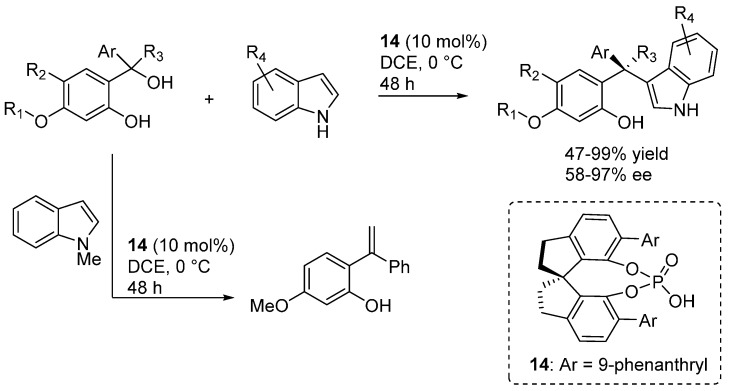
Addition of indoles *o*-QMs delivering products with quaternary stereocenters.

Another highly relevant extension of the *o*-QM structures which can be engaged in this type of Brønsted acid catalyzed reactions was reported by Sun, who demonstrated that tertiary benzhydryl alcohols react with indoles in the presence of catalyst **14** delivering products bearing a challenging all-carbon quaternary stereocenter (Scheme 21) [30]. The reaction appeared limited to electron-rich phenols, and the intermediacy of an *o-*QM intermediate, which structure was more thoroughly elucidated in a subsequent work (see Section 3.3), was suggested by several control experiments. Since *N*-methyl indole furnished exclusively a styrene elimination product, in this case the usual double activation furnished by the acid through coordination at the indole N-H was also invoked. Curiously, the same styrene side-product was also observed when some non-optimal phosphoric acid catalysts were applied to the reaction. Considering the results reported in Section 3.3, it is not clear whether this styrene is an intermediate of the reaction or a dead-end side-product.

**Scheme 22:**
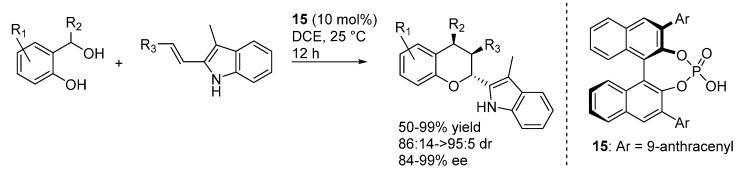
Inverse electron demand [4 + 2] oxa-Diels-Alder of *o*-QMs with 2-vinylindoles catalyzed by phosphoric acid **15**.

The coordination of these phosphoric acid catalysts to the indole N-H was exploited in a different reaction by Shi, who employed the activated olefin of 2-vinyl-3-methyl substituted indoles in a [4 + 2] inverse electron demand oxa-Diels-Alder reaction with *o*-QMs catalyzed by **15** (Scheme 22) [31]. A substituent at the 3-position of the indole was found to be mandatory for the reaction to proceed through the cycloaddition pathway, instead of the simple addition of the indole (see e.g., Scheme 14 and Scheme 18). Furthermore, *Z*-vinylindoles were found to isomerize to their more stable (and more reactive towards the cycloaddition) *E*-isomers under the reaction conditions, thus allowing using *E*/*Z* mixtures in the reactions, which were assumed to occur through a concerted pathway. Remarkably and in contrast with most of the examples highlighted so far, the reaction worked well with substrates bearing not only aryl but also alkyl substituents at the benzylic position (*i.e.*, R_2_ in Scheme 22).

**Scheme 23:**
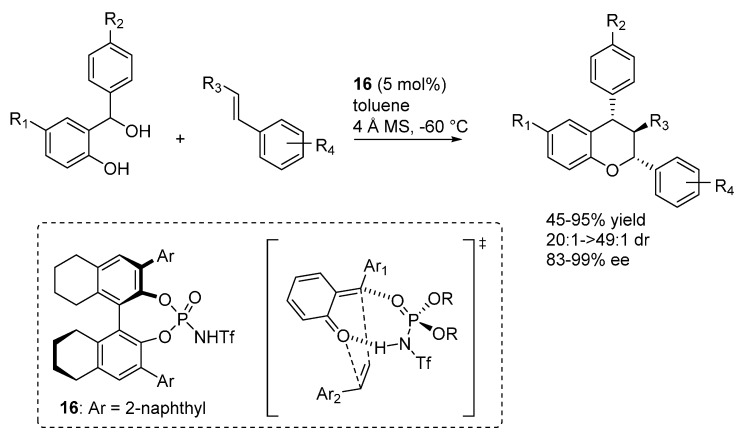
Inverse electron demand [4 + 2] oxa-Diels-Alder reaction of *o*-QMs with styrenes catalyzed by phosphorimide **16**.

A different hetero-Diels-Alder reaction of *o*-QMs involving styrenes as dienes was reported by Rueping (Scheme 23) [32]. This reaction presents some very challenging aspects, related to the low nucleophlicity of styrenes, to the tendency of these olefins to undergo polymerization under acidic reaction conditions, and especially to their lack of an anchor for catalyst coordination. In fact, the reaction represents one of the rare examples wherein nucleophile coordination does not seem to be possible, in contrast with the usual bifunctional activation mode expressed by phosphoric acid and related catalysts, also seen in the previous examples highlighted in this section. To account for the excellent enantioselectivites observed when the phosphorimide catalyst **16** was employed, an open transition state model involving bicoordination of the phosphorimide to the *o*-QM was proposed. The formation of such a complex, wherein the phosphorimide coordinates the *o*-QM oxygen with its acidic proton and the electropositive exocyclic olefin carbon with its Lewis basic oxygen, was also supported by NMR experiments.

### 3.2. o-QMs Generated in Situ by Alcohol Elimination from Ortho-Hydroxybenzylic Ethers under Lewis Acid Conditions

**Scheme 24:**
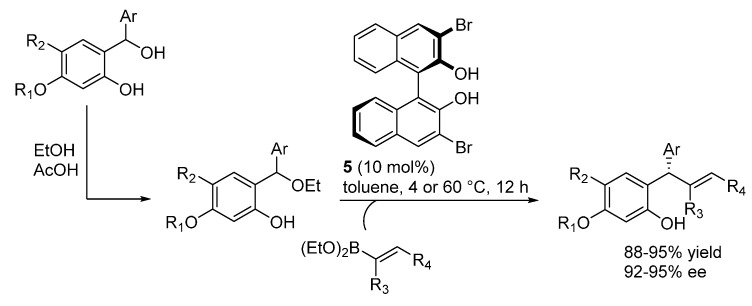
Addition of vinyl boronates to *o*-QMs generated from *ortho*-hydroxybenzylic ethers.

In the frame of their studies on catalytic asymmetric boronate additions to *o*-QMs promoted by chiral binaphthols, summarized in Section 2 (Scheme 10), Luan and Schaus extended the reaction to less stable *o*-QMs through their generation *in situ* [16]. Whereas *ortho*-hydroxybenzylic alcohols did not give optimal results as *o*-QMs precursors, the corresponding more stable ethyl ethers, readily prepared in acidic ethanol, proved to be more suitable (Scheme 24). Under conditions essentially identical to the ones employed with pre-formed *o*-QMs, a range of ethers could be applied to the reaction (Scheme 24). Its scope was thus considerably extended, although an electron donating ether substituent at the phenol ring was still required.

**Scheme 25:**
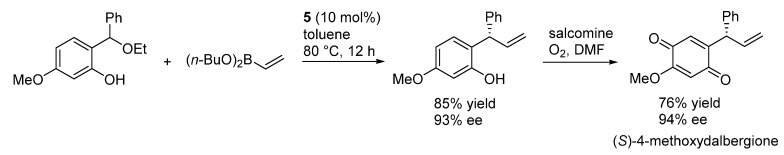
Addition of vinylboronate and synthesis of (*S*)-4-methoxydalbergione.

By applying more forcing conditions (80 °C), even the simple vinyl boronate could be employed as nucleophile in the reaction, delivering a product that could easily be transformed into the natural product (*S*)-4-methoxydalbergione (Scheme 25).

### 3.3. o-QMs Generated in Situ by 1,6-H Shift of Ortho-Hydroxystyrenes under Brønsted Acid Conditions

**Scheme 26:**
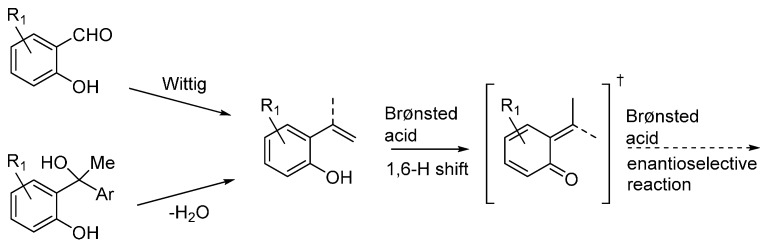
Preparation of *ortho*-hydroxystyrenes and reactions under acidic conditions.

The possibility of using *ortho*-hydroxystyrenes as *o*-QMs precursors in organocatalytic asymmetric reactions with nucleophiles has been reported by three laboratories almost simultaneously in 2015 [33,34,35]. All three reports dealt with chiral phosphoric acid catalysts, providing protocols alternative to the synthetic platform developed by Sigman giving *o*-QM through palladium-hydride initiated H-shifts from these styrenes [11]. The *ortho*-hydroxystyrenes substrates can be prepared by Wittig olefination of the corresponding salicylaldehydes, or by dehydration of suitable tertiary alcohols (Scheme 26). The phosphoric acid catalysts are then able to promote a 1,6-H shift by protonating the electron-rich olefin while abstracting the phenolic proton (see discussion below).

**Scheme 27:**
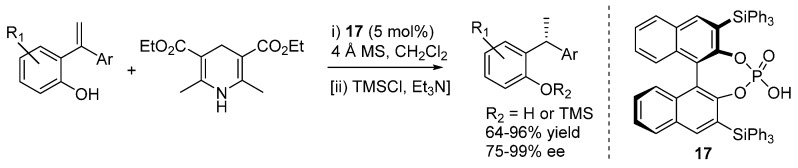
Transfer hydrogenation of 2-hydroxystyrenes with Hantzsch ester catalyzed by phosphoric acid **17**.

Sun *et al.* used α,α-disubstituted styrenes and described the transfer hydrogenation reaction with Hantzsch esters as hydride donors, delivering unsymmetrically substituted 1,1-diarylethanes with very good results under the action of catalyst **17** (Scheme 27) [33]. Six randomly selected compounds were tested for cytotoxicity against human cancer cell lines, and one of them displayed remarkable activity. In some cases, it was found to be convenient to isolate the products as the corresponding trimethylsilyl ethers.

**Scheme 28:**
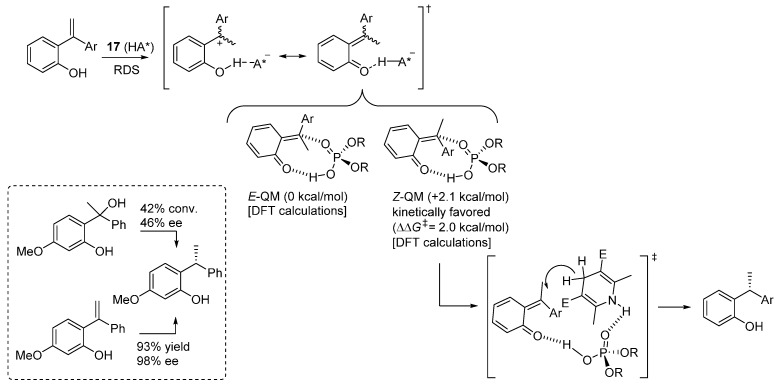
Proposed reaction pathway and control experiment with the benzylic alcohol in the transfer hydrogenation reaction of 2-hydroxystyrenes with Hantzsch esters.

Substantiated by several control experiments, the proposed reaction pathway involves interaction of the electron rich styrene olefin with the acidic catalyst, resulting in a species which can be represented by two limiting resonance structures, a zwitterionic and a neutral (*o*-QM-like) one (Scheme 28). DFT (B3LYP-D3) calculations pinpointed that the neutral structure, bicoordinated to the catalyst, is a more accurate representation of the electronic distribution of this intermediate, and that formation of the less stable *Z*-QM is kinetically favored over its *E*-counterpart. Kinetic experiments showed that the reaction is zeroth order in Hantzsch ester. Therefore, *o*-QM generation is the rate determining step, and the reaction is likely to occur via the addition of the Hantzsch ester to the less stable *Z*-QM intermediate, through a stereodetermining transition state involving the usual bifunctional action of the catalyst, which coordinates the *o*-QM with its acidic proton and the Hantzsch ester NH with the phosphoryl oxygen. An interesting control experiment, wherein a benzylic alcohol as *o*-QM precursor gave dramatically reduced results compared to the *ortho*-hydroxystyrene, demonstrated the importance of the strategy employed for the *o*-QM generation, especially for the control of its geometry and thus ensuing stereoselectivity in the following addition step.

**Scheme 29:**
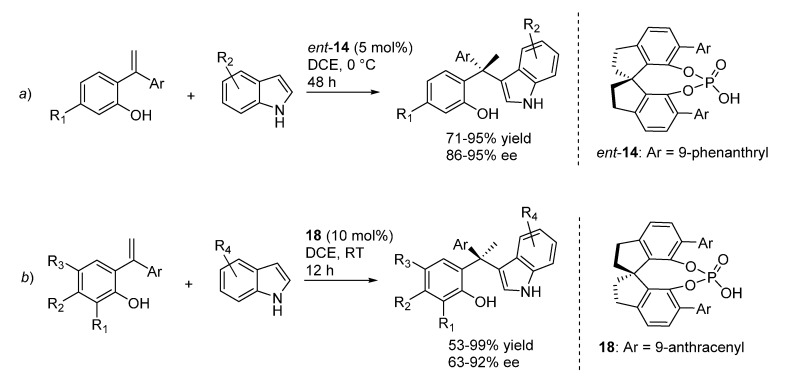
Catalytic asymmetric additions of indoles to *o*-QMs generated *in situ* from *ortho*-hydroxystyrenes reported by Sun *et al.* (**a**, [33]) and by Wu *et al.* (**b**, [34]).

Despite these latter considerations, the same catalyst **14** previously applied by the same laboratory to the addition of indoles to *o*-QMs generated from benzylic alcohols (Scheme 21), proved to also be an excellent promoter when the *o*-QMs were generated from *ortho*-hydroxystyrenes (Scheme 29a), indicating perhaps a change in the RDS between the transfer hydrogenation and the Friedel-Crafts reactions. Some of the previously highlighted limitations of the Friedel-Crafts reaction (the requirement of an ether substituent at the phenol ring to stabilize the *o*-QM and assist its formation) were somehow alleviated with this new protocol, which, however, still necessitated the presence of an electron releasing group in at least one of the two aryl substituents of the styrenes. This latter limitation is also present in a similar protocol for the same transformation, reported almost simultaneously by Wu *et al.*, which is based on a closely related catalyst **18** and includes a more thorough study of the reaction scope (Scheme 29b) [34].

**Scheme 30:**
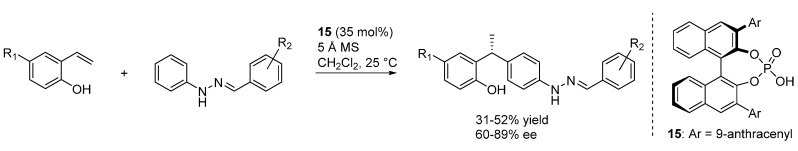
Catalytic asymmetric additions of hydrazone activated aryls to *o*-QMs generated *in situ* from *ortho*-hydroxystyrenes catalyzed by phosphoric acid **15**.

A distinct transformation based on the generation of *o*-QM intermediates from *ortho*-hydroxystyrenes was reported by Shi [35]. In contrast with the above examples involving α,α-disubstituted *ortho*-hydroxystyrenes, in this case simpler α-monosubstituted substrates were used for the reaction, a hydroarylation of the *o*-QM promoted by the chiral Brønsted acid catalyst **15** at high loadings (Scheme 30). A hydrazone served as an activating unit, rendering aryl rings sufficiently nucleophilic at their *para*-position to undergo the asymmetric additions, although the products were obtained with moderate results. A transition state involving two catalyst units, one activating the *o*-QM and the other coordinating the remote hydrazone was proposed.

Furthermore, as we recently reviewed [36], the intermediacy of *o*- and *p*-QMs has been invoked in some Brønsted acid catalyzed Povarov and related cycloaddition reactions, wherein addition of hydroxystyrenes to acid activated electrophiles brings about the formation of a QM undergoing an intramolecular conjugate addition, resulting in formal cycloadditions between the activated styrene olefin and various dienes.

### 3.4. o-QMs Generated in Situ by Desilylation—Halide Elimination from Ortho-Silyloxy Benzylic Halides under Lewis Basic Conditions

**Scheme 31:**
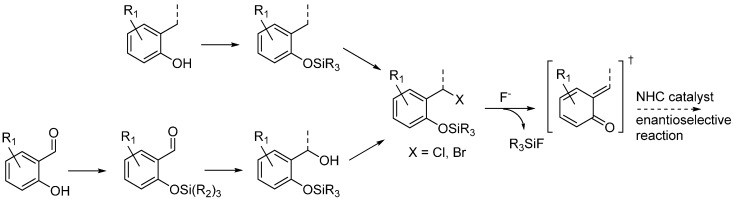
Preparation of *O*-silyl phenols bearing a leaving group at the benzylic position, and ensuing organocatalytic reaction.

*O*-Silyl protected phenols bearing a leaving group at the benzylic position, such as an halide, can be employed for the generation of *o*-QMs *in situ* under the promotion of Lewis bases (typically fluorides) (Scheme 31). These substrates were introduced to overcome the poor stability of the corresponding free phenols, and to guarantee a ionic control over the *o*-QM generation [37]. Their preparation entails silyl protection of the phenol followed by either radical halogenation [38] or hydroxide substitution [39], depending on the phenolic structure employed. This method of *o*-QM formation has been successfully exploited in asymmetric organocatalysis by Scheidt, by flanking the stoichiometric fluoride Lewis base, used to generate the *o*-QM, with chiral Lewis basic catalysts able to combine selectively with the nucleophilic component: *N*-heterocyclic carbenes (NHCs) [40,41].

**Scheme 32:**
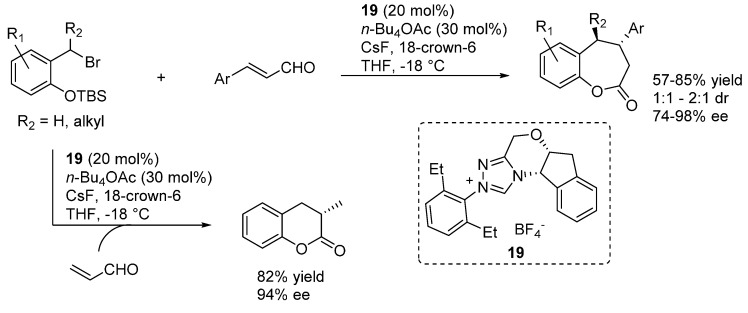
Formal [4 + 3] cycloaddition reaction of cinnamaldehydes with *o*-QMs catalyzed by NHC generated from precursor **19**.

In more detail, two formal cycloaddition reactions were developed exploiting this strategy, where a substantial optimization process was required to find conditions suitable for combining the desilylation-elimination step with the NHC catalyzed reactions. The first report described a formal [4 + 3] cycloaddition with cinnamaldehydes, and employed cesium fluoride/18-crown-6 as fluoride source, with tetra-*n*-butylammonium acetate as a mild Brønsted base to generate the NHC catalyst from pre-catalyst **19** (Scheme 32) [40]. While chloride and bromide could be equally used as leaving groups, the choice of the silyl substituents proved to be crucial, with robust moieties such as TIPS and TBS allowing a controlled generation of the *o*-QM and giving much better results than more labile groups such as TES. Remarkably, also β-unsubstituted, and thus highly unstable, *o*-QMs could participate in the reaction. Whereas cinnamaldehydes followed the [4 + 3] cycloaddition pathway, aliphatic enals or acrolein delivered a [4 + 2] cycloaddition product.

**Scheme 33:**
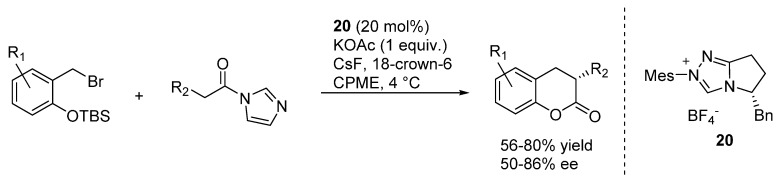
[4 + 2] cycloaddition reaction of *N*-acylimidazoles with *o*-QMs catalyzed by NHC generated from precursor **20**.

A related [4 + 2] cycloaddition reaction was indeed the subject of the subsequent publication, wherein *N*-acylimidazoles were applied as carbonyl donors in combination with pre-catalyst **20** (Scheme 33) [41]. To avoid racemization of the obtained 3,4-dihydrocoumarins, it turned out to be necessary to change the weak Brønsted base used for catalyst generation. Eventually, by swapping the previously employed tetra-*n*-butyl ammonium acetate with its potassium salt, it turned out to be possible to obtain adducts with moderate to good enantioselectivities. These transformations well complement the related NHC-catalyzed transformations reported by Ye and highlighted in Section 2, wherein pre-formed *o*-QMs were employed.

**Scheme 34:**
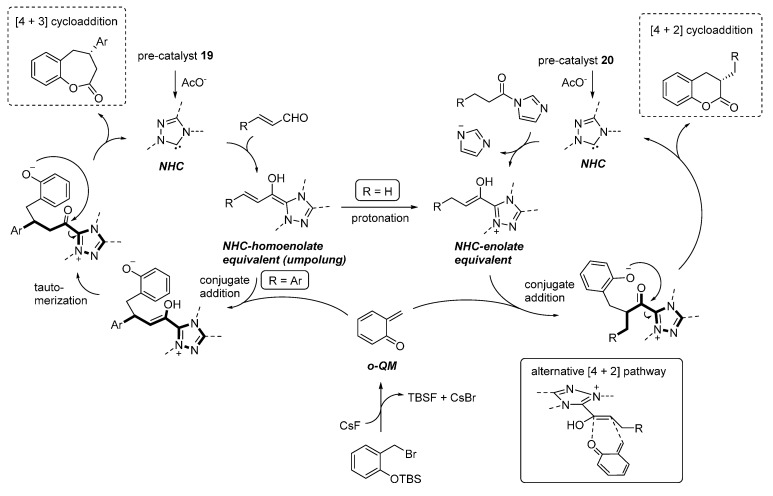
Reaction pathways followed by the cycloaddition reactions catalyzed by NHCs.

The [4 + 3] and [4 + 2] cycloadditions were proposed to follow related reaction pathways, involving the generation of the reactive *o*-QM by the action of fluoride (Scheme 34). In the case of enals reaction partners, their simultaneous combination with the NHC catalyst, obtained by deprotonation of precatalyst **19**, brings about an *umpolung* of these substrates by forming an NHC homoenolate equivalent. Here, the fate and the reactivity of this species depends on the enal involved. The NHC-homoenolates derived from cinnamaldehydes add to the *o*-QM in a conjugate fashion. Upon tautomerization, the phenoxide displaces the NHC catalyst ensuring completion of the catalytic cycle and formation of a formal [4 + 3] cycloaddition product. Conversely, the NHC-homoenolate derived from acrolein undergoes protonation at the terminal position faster than the conjugate addition, giving an NHC enolate equivalent and ultimately resulting in a [4 + 2] cycloaddition. Related NHC-enolates are produced directly from *N*-acylimidazoles and the NHC catalyst derived from **20**. As an alternative to the sequential conjugate addition phenoxide displacement pathway, a concerted [4 + 2] cycloaddition between the *o*-QM and the NHC enolate was also considered to be possible.

### 3.5. o-QMs Generated in Situ by Desilylation—Halide Elimination from Ortho-Silyloxy Benzylic Halides under Brønsted Acid Conditions

**Scheme 35:**
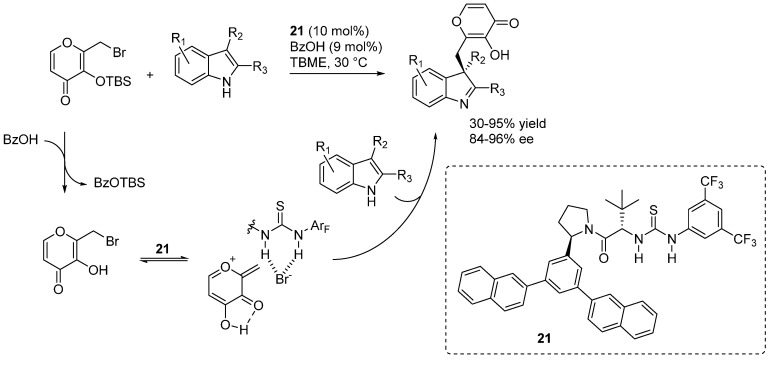
Addition of indoles to a pyrone derived *o*-QM catalyzed by chiral thiourea **21** and achiral BzOH Brønsted acids.

During their studies directed at the synthesis of pleiomaltinine, Porco and coworkers reported that acidic conditions are more effective than usual fluoride treatment in generating an *o*-QM-like intermediate from an *O*-TBS pyrone, and that this *o*-QM species undergoes indole additions [42]. In a collaborative effort with the Jacobsen laboratory, this synthetic transformation was implemented into its catalytic enantioselective version, wherein the combination of chiral thiourea **21** and achiral Brønsted acid (BzOH) catalysts promotes enantioselective additions of 3-substitued indoles to a pyrone-derived *o*-QM (Scheme 35) [43]. Whereas the reaction was found to be essentially unsensitive to the achiral acid co-catalyst used, the leaving group on the pyrone had a pronounced effect. Furthermore, the presence of the indole N-H was essential for enantioselectivity. On these grounds, a reaction pathway was proposed. Achiral Brønsted acid promoted desilylation delivers a pyrone, which suffers elimination of its leaving group forming an *o*-QM-like cationic intermediate. Coordination of the leaving group to the thiourea catalyst **21** not only favors its elimination but also provides a chiral environment around the reactive cationic intermediate. The stereodetermining step, the addition of the indole to the thus coordinated intermediate, proceeds through concomitant general base catalysis. The importance of the large *m-*terphenyl group on the catalyst strongly suggests that π-interactions exert a key role in controlling the stereochemistry of the addition.

### 3.6. o-QMs Generated in Situ by Sulfinic Acid Elimination from 2-Sulfonylalkyl Phenols under Brønsted Basic Conditions

**Scheme 36:**
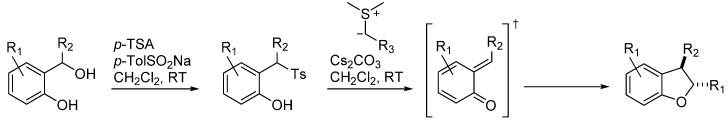
Preparation of 2-sulfonylalkyl phenols and their use as *o*-QM precursors.

A straightforward approach to *o*-QMs generation is the base induced elimination of a leaving group from the benzylic position of suitably functionalized phenols. However, the aforementioned poor stability of *ortho*-hydroxybenzylic halides prevented this approach from being fully adopted and exploited, with silyl protection at the phenolic oxygen, to be removed under Lewis basic conditions (see Section 3.4), being the most pursued alternative strategy. This stability issue was circumvented only very recently, by applying a leaving group different from a halide, namely an arylsulfonyl moiety. Inspired by the strategic employment of arylsulfonyl moieties to temporarily trap relatively unstable intermediates such as alkylideneindolenines [44] and *N*-carbamoyl imines [45], Zhou reported in 2013 the straightforward preparation of 2-arylsulfonylalkyl phenols from the corresponding alcohols and their employment in a synthesis of 2,3-benzofurans, wherein *o*-QMs intermediates were generated from these sulfonyl species under mild Brønsted basic reaction conditions (Scheme 36) [46]. The same strategy could also be applied to the synthetically appealing additions of cyanide [47] and ammonia [48].

These reports paved the way to the application of chiral Brønsted basic catalyst for activation of *o*-QMs reaction partners. Despite its considerable synthetic utility, employment of chiral Brønsted bases results unfeasible with the other methodologies for *o*-QMs generation described in Section 3.1, Section 3.2, Section 3.3, Section 3.4 and Section 3.5. Whereas a low, enantioselective example restricted to one substrate was first reported by Zhou in the frame of the addition of ammonia [48], two papers, appearing almost simultaneously, demonstrated that high enantioselectivity can be achieved in this approach, and highlighted relevant challenges.

**Scheme 37:**
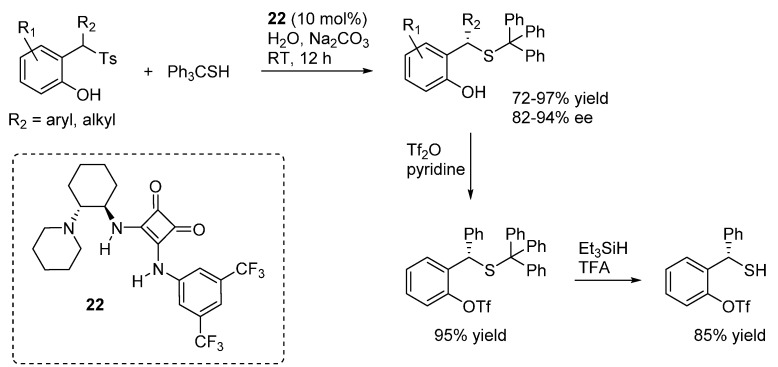
Catalytic asymmetric additions of tritylthiol to *o*-QMs catalyzed by **22**.

Liu and Li reported the addition of tritylthiol to *o*-QMs generated from 2-tosylalkyl phenols (Scheme 37), promoted by the bifunctional organocatalyst **22** and proceeding in the absence of organic solvents (just a small amount of dichloromethane was added to dissolve the substrates) [49]. Aqueous sodium carbonate was used as the stoichiometric inorganic base to neutralize the sulfinic acid formed in the reaction. It was suggested that spatial separation between the stoichiometric inorganic base and the chiral organic base **22** was the key in achieving excellent enantioselectivities. Remarkably, the reaction could be applied successfully not only to β-aryl *o*-QMs but also to their less stable β-alkyl counterparts, whereas limitations appeared with more stabilized *o*-QMs (*i.e.*, R_1_ = electron releasing substitutent in Scheme 37). Furthermore, deprotection of the thiols was demonstrated to be feasible after phenol triflation, thus giving an entry to otherwise difficult to access benzyl thiols in highly enantioenriched form.

**Scheme 38:**
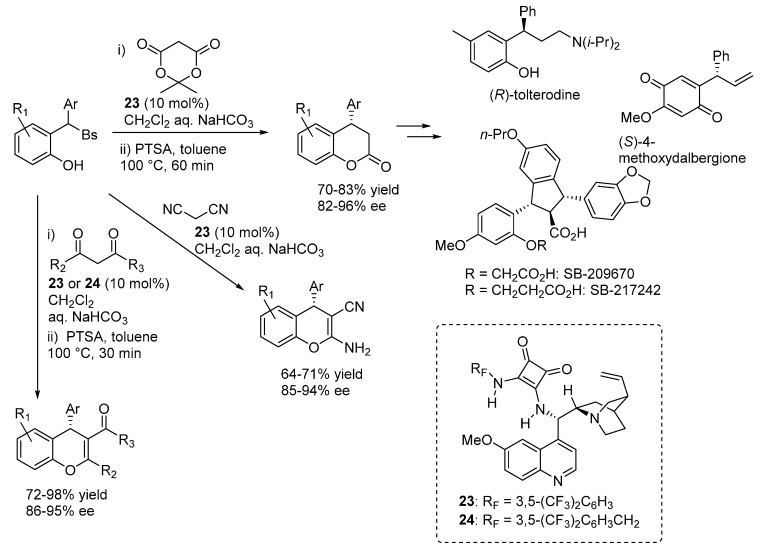
Catalytic asymmetric addition of 1,3-dicarbonyl compounds to *o*-QMs catalyzed by **23** and **24**.

Nearly at the same time, our laboratory reported on the addition of various 1,3-dicarbonyl compounds (Meldrum’s acid, malononitrile, 1,3-diketones and 3-ketoesters) to *o*-QMs generated *in situ* from 2-sulfonylalkyl phenols, proceeding under the combined action of a stoichiometric inorganic base (aq. NaHCO_3_, used in large excess) and the bifunctional catalysts **23** and **24** (Scheme 38) [50]. Key to success was the discovery that a subtle tuning of the leaving group properties of the sulfonyl moiety, using a less electron rich phenylsulfone instead of the usual *p*-tolyl one, had a profound impact on the reaction outcome. Cyclizations, sometimes ensured by dehydrative acidic treatment after the catalytic step, followed the conjugate additions, delivering a range of 3,4-dihydrocoumarins and 4*H*-chromenes with a fully complementary scope with respect to the catalytic additions of 1,3-diketones highlighted in Section 3.1 and proceeding under acidic conditions. Some of the 3,4-dihydrocoumarin adducts obtained from the reactions with Meldrum’s acid are known intermediates for the synthesis of tolterodine, the active pharmaceutical ingredient of the antimuscarinic drug Detrol, and the endothelin antagonists SB-209670 and SB-217242, whereas another compound was converted in a synthetic precursor of the natural compound (*S*)-4-methoxydalbergione. Both electron releasing and withdrawing substituents could be installed at the phenol ring, however the reaction appeared limited to β-aryl *o*-QMs.

**Scheme 39:**
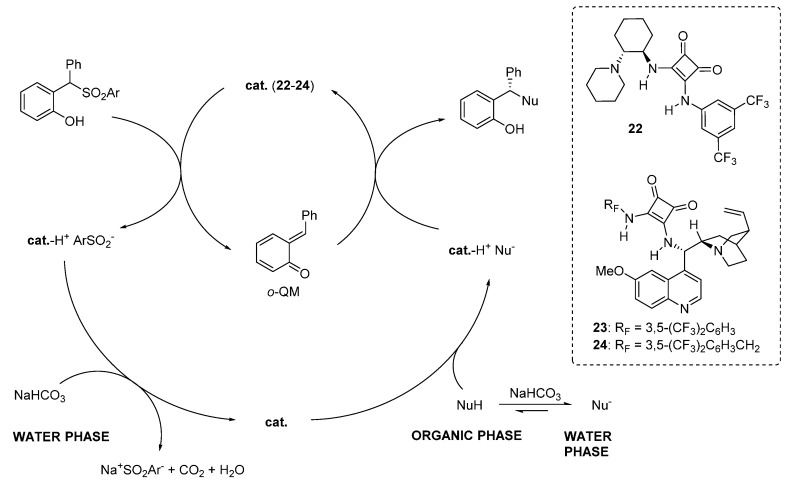
Proposed reaction pathway for catalytic asymmetric additions to *o*-QMs generated *in situ* from 2-sulfonylalkyl phenols.

A nearly identical reaction pathway was proposed in the two papers, wherein the chiral organic base is responsible not only for the asymmetric addition, but also for the generation of the *o*-QM by deprotonating the phenol (Scheme 39). Thus, the role of the inorganic base is to regenerate the catalyst in its active form (the free amine), allowing the reaction to proceed. Whereas catalyst regeneration is the rate determining step of the overall catalytic cycle, it was determined that at least in some cases a large part of the nucleophile reaction partners are in the aqueous phase, due to their considerable acidity. Despite this unfavorable partition, both reactions appeared highly efficient indicating perhaps the requirement of a substantial amount of free catalyst (*i.e.*, not complexed with the pro-nucleophiles) in the organic phase for *o*-QM generation.

## 4. Catalytic Asymmetric Reactions with *p*-QMs

*para*-Quinone methides (*p*-QMs) are structural isomers of *o*-QMs, displaying a cyclohexadiene core with a carbonyl group and an exocyclic alkylidene residue placed at the *para* position. Also, *p*-QMs exist in a variety of natural products, pharmaceuticals [51,52], and can be found as reactive intermediates in many chemical and biological processes [53]. The synthesis and the reactivity of *p*-QMs depend on the nature of the electron-donating substituents installed on the cyclohexadiene core. Although electron-donating groups are mandatory for their isolation, compared to *o*-QMs, less strong EDG are required [54], resulting in a broader scope of using stabilized *p*-QMs. The aromatization of the cyclohexadiene ring constitutes the driving force of the 1,6-conjugated addition of nucleophiles to *p*-QMs, rendering these substrates attractive target, due to the possibility to rapidly afford important chiral diarylmethines stereocenters.

**Scheme 40:**
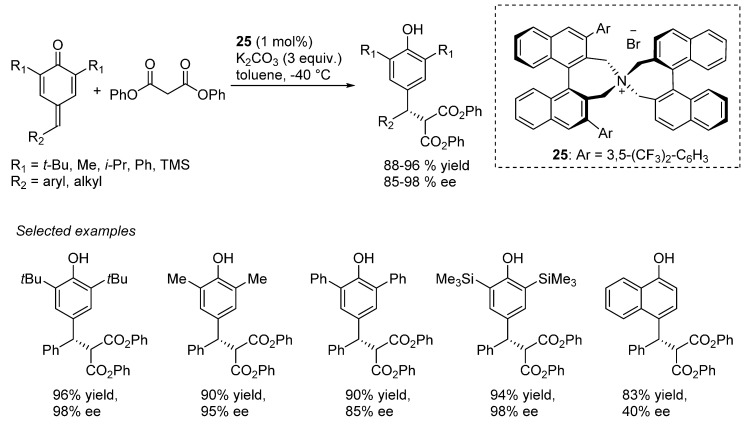
Asymmetric 1,6-conjugated addition of malonates to *p*-QMs under phase-transfer conditions.

The first report concerning an organocatalytic transformation of *p*-QMs was presented by Fan in 2013, describing the asymmetric synthesis of diarylalkanes through a catalytic enantioselective 1,6-conjugated addition of malonates to *p*-QMs, under phase-transfer catalysis (Scheme 40) [55]. The catalytic reaction of preformed and stabilized *p-*QMs was promoted by the axially chiral binaphthyl-modified catalysts **25** in the presence of potassium carbonate as inorganic base, while, as nucleophilic counterpart diphenyl malonate performed better amongst a series of related esters. To evaluate the scope of this asymmetric methodology, a broad series of *p*-QMs were synthetized and employed in the catalytic transformation. The reaction of *p*-QMs in which R_1_ = *t-*Bu was found to be tolerant not only to a series of electron-neutral, electron-deficient and electron-rich aryls at R_2_, but also to aliphatic residues at the same side chain, providing, in most of cases, excellent enantioselectivities and high yields. In addition to the above investigation, *p*-QMs bearing non-*tert*-butyl stabilizing group at R_1_ were also probed for the asymmetric 1,6-conjugated addition: methyl, *iso-*propyl, phenyl and trimethylsilyl substituents gave analogous good results. Interestingly, a *p*-QM derived from 1,4-naphthoquinone was described, albeit only a moderate enantiomeric excess was obtained.

**Scheme 41:**
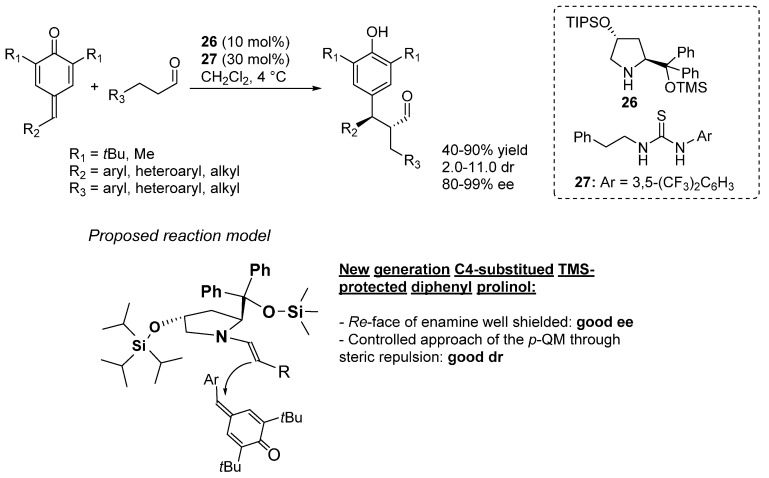
Organocatalytic α-alkylation of aldehydes via 1,6-conjugated addition of enamine-activated aldehydes to *p*-QMs.

In 2014, Jørgensen described a novel approach for the α-alkylation of aldehydes by employing *p*-QMs as alkylating agents under enamine catalysis [56]; remarkably, this methodology afforded α-diarylmethine substituted aldehydes with two contiguous stereocenters in high yields and excellent stereoselectivities (Scheme 41). Although the commonly employed *O*-TMS diphenyl prolinol catalyst gave the α-alkylated products in good yield and enantioselectivity, the diastereoselectivity of the reaction remained poor. In order to take on this challenge, a new secondary amine catalyst **26** was designed: here, the (diphenylmethyl)trimethylsiloxy group was flanked by another bulky silyloxy moiety, placed at the C4-position of the pyrrolidine ring and in *trans* relationship to the C1-substituent. Whereas the (diphenylmethyl)trimethylsilyloxy group provided for the enantio-control, the bulky triisopropylsilyloxy group was the key to sterically determine the approach of *p*-QMs to the enamine-activated aldehydes, hence controlling the diastereoselectivity. Moreover, by using the thiourea **27** as co-catalyst, the yield of the reaction was further improved, possibly by activation of the carbonyl group of the *p*-QMs through hydrogen bonding. The scope of the reaction was then investigated by employing a series of *p*-QMs bearing electron-rich, electron-neutral and electron-poor aromatic moieties in the reaction with hydrocinnamaldehyde, affording the corresponding products in good yields and high diastereo- and enantioselectivities. Also heteroaromatic substituted *p*-QMs reacted smoothly under these conditions, while the *p*-QM in which R_2_ is a methyl group gave the corresponding product only in moderate yield and stereoselectivity. However, replacing the *tert*-butyl substituents R_1_ for methyl group did not change the yield and stereoselectivity of the reaction significantly. Finally, the aldehyde scope was also explored: aryl, heteroaryl and alkyl substituents in the aldehyde side-chain (R_3_) were well tolerated.

**Scheme 42:**
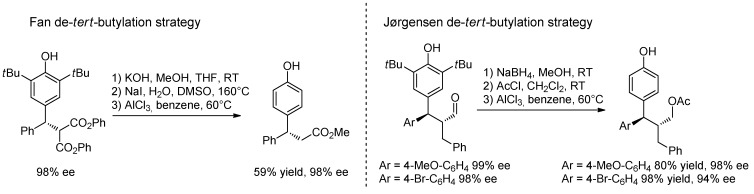
Elimination of the *tert*-butyl group from the phenolic ring of the catalytic products derived from asymmetric additions to *p*-QMs.

In most cases, Fan and Jørgensen used 2,6-di-*tert*-butyl *p-*QMs due to their facile synthesis and inherent stability. Notably, the *tert*-butyl groups on the phenolic ring could be removed afterwards by treatment with AlCl_3_ without loss of stereoinformation (Scheme 42).

As summarized in Section 3.3 (Scheme 27 and Scheme 28), Sun and co-workers described an organocatalytic transfer hydrogenation for the asymmetric synthesis of 1,1-diarylethanes [33]. The reaction was found to proceed with chiral phosphoric acid promoted 1,6-H shift on *o*-hydroxystyrenes, delivering *o*-QM intermediates, which captured the hydride from Hantzsch ester and gave the corresponding 1,1-diarylalkanes bearing *o*-hydroxyphenyl units with excellent efficiency and enantioselectivity.

**Scheme 43:**
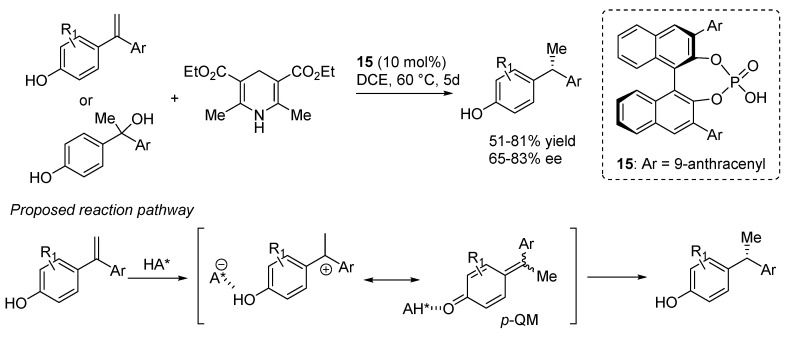
Asymmetric synthesis of 1,1-diarylethanes by transfer hydrogenation, proceeding through *in situ* generated *p*-QMs.

Remarkably, the authors extended the protocol to substrates bearing a *p*-hydroxy instead of an *o*-hydroxy directing group. After extensive re-optimization of the reaction conditions, it was identified that with phosphoric acid **15**, α,α-diarylolefin and benzhydryl racemic tertiary alcohols having *p*-hydroxy group could smoothly react to form the target hydrogenation products in variable yields and acceptable enantioselectivities (Scheme 43). Albeit the stereoinductions of the reactions were reasonably good, the absence of an *ortho*-hydroxyl group, as a close anchoring point for the catalyst, had negative effects for the enantioselectivities that were in all cases lower compared to the parent *o*-hydroxyl substrates. It was believed that the reaction proceeded via the generation of a transient *p*-QM species arisen upon H-shifts in the *p*-hydroxy styrene substrates.

## 5. Conclusions

In a very short time, QMs have changed their status from being exotic intermediates, nearly unseen in asymmetric catalytic reactions, to useful and widely exploited substrates combined with different nucleophilic partners under the promotion of organic catalysts. Most attention has been devoted to *o*-QMs, wherein several useful protocols encompassing their generation *in situ* for organocatalytic asymmetric reactions are now available. However, several attractive methods for *o*-QMs generation *in situ* (e.g., through oxidation) are still “orphan” of a chiral catalyst combination. Whereas it seems likely that the available methodologies will find use in the frame of natural product synthesis, structurally varied *o*-QMs, such as their aza-counterpart, can also beneficiate from the strategies developed so far, as witnessed by recent works disclosing useful catalytic asymmetric reactions to nitrogen containing benzo-fused heterocycles based on aza-analogues of *o*-QMs [57,58]. Furthermore, the chemistry of the *p*-QMs has recently begun to emerge, introducing these extended enones as “new players” in asymmetric catalysis [59].
